# Developing non-response weights to account for attrition-related bias in a longitudinal pregnancy cohort

**DOI:** 10.1186/s12874-023-02121-1

**Published:** 2023-12-14

**Authors:** Tona M. Pitt, Erin Hetherington, Kamala Adhikari, Shainur Premji, Nicole Racine, Suzanne C. Tough, Sheila McDonald

**Affiliations:** 1 Department of Paediatrics, University of Calgary, 28 Oki Drive NW, Calgary, T3B 6A8 Canada; 2 Department of Epidemiology, Biostatistics and Occupational Health, McGill University, 2001 McGill College, Montreal, H3A 1G1 Canada; 3 Department of Community Health Sciences, University of Calgary, 3280 Hospital Drive NW, Calgary, T2N 4Z6 Canada; 4 Provincial Population and Public Health, Alberta Health Services, 10301 Southport Rd SW, Calgary, T2W 1S7 Canada; 5 Centre for Health Economics, University of York, Heslington, YO10 5DD York UK; 6 School of Psychology, Faculty of Social Sciences, University of Ottawa, 136 Jean- Jacques Lussier, Ottawa, K1N 6N5 Canada; 7 Children’s Hospital of Eastern Ontario Research Institute, 401 Smyth Rd, Ottawa, K1H 5B2 Canada

**Keywords:** Cohort studies, Inverse probability weights, Non-response weights, Attrition, All our families

## Abstract

**Background:**

Prospective cohorts may be vulnerable to bias due to attrition. Inverse probability weights have been proposed as a method to help mitigate this bias. The current study used the “All Our Families” longitudinal pregnancy cohort of 3351 maternal-infant pairs and aimed to develop inverse probability weights using logistic regression models to predict study continuation versus drop-out from baseline to the three-year data collection wave.

**Methods:**

Two methods of variable selection took place. One method was a knowledge-based a priori variable selection approach, while the second used Least Absolute Shrinkage and Selection Operator (LASSO). The ability of each model to predict continuing participation through discrimination and calibration for both approaches were evaluated by examining area under the receiver operating curve (AUROC) and calibration plots, respectively. Stabilized inverse probability weights were generated using predicted probabilities. Weight performance was assessed using standardized differences of baseline characteristics for those who continue in study and those that do not, with and without weights (unadjusted estimates).

**Results:**

The a priori and LASSO variable selection method prediction models had good and fair discrimination with AUROC of 0.69 (95% Confidence Interval [CI]: 0.67–0.71) and 0.73 (95% CI: 0.71–0.75), respectively. Calibration plots and non-significant Hosmer-Lemeshow Goodness of Fit Tests indicated that both the a priori (p = 0.329) and LASSO model (p = 0.242) were well-calibrated. Unweighted results indicated large (> 10%) standardized differences in 15 demographic variables (range: 11 − 29%), when comparing those who continued in the study with those that did not. Weights derived from the a priori and LASSO models reduced standardized differences relative to unadjusted estimates, with the largest differences of 13% and 5%, respectively. Additionally, when applying the same LASSO variable selection method to develop weights in future data collection waves, standardized differences remained below 10% for each demographic variable.

**Conclusion:**

The LASSO variable selection approach produced robust weights that addressed non-response bias more than the knowledge-driven approach. These weights can be applied to analyses across multiple longitudinal waves of data collection to reduce bias.

## Introduction

Longitudinal study designs allow researchers to establish temporality of exposure-outcome relationships by following samples of individuals over time with repeated measurements [1]. It is, however, common for participants in longitudinal cohorts to be lost to follow-up (i.e., attrition or censoring) [2]. While attrition over time is expected, it can contribute to biased exposure-outcome relationships depending on the nature of how and/or why individuals drop out of the study [3]. Attrition leaves researchers with challenges on how to address missing data, which will depend on why the data are missing, and has implications for analysis.

Several methods exist that aim to mitigate potential bias related to attrition. Complete case analysis and multiple imputation are used commonly, but both rely on assumptions related to how data are missing [4]. Attrition contributes to missing data that may be missing at random, missing not at random, or missing completely at random. Each changes the assumptions of how data are missing and the potential bias that may occur if one were to apply a complete case analysis [1, 4]. Missing at random values are conditional on observed data, missing not at random depends on unobserved data, and missing completely at random depends on neither [1, 4]. Another way to address attrition/censoring is to weight existing respondents using inverse probability of participation weights that are calculated based on the baseline information [5–7]. That is, the inverse of the probability of continuing in the study at subsequent waves of collection (i.e., those who have lower probability of continuing receive higher weights). This method accepts that individuals may drop out of longitudinal studies for various reasons and that these reasons can be modelled through weighting and using the existing data.

This study aimed to describe the process for developing and assessing the performance of weights in a pregnancy cohort that has spanned approximately 14 years to provide a statistical approach to account for attrition and the potential for selection bias. We describe two methods for developing a model to create the weights (one knowledge-based a priori model that is investigator derived and another data driven model using Least Absolute Shrinkage and Selection Operator [LASSO] regression), assessed the discrimination and calibration performance of each model, and then assessed the performance of the weights from each model. The best performing model was then applied to subsequent waves of data collection, and the performance of these weights was assessed to consider using these weights across all data collection waves in this cohort.

## Methods

### Cohort description

This study used the data from All Our Families Cohort (formerly All Our Babies Cohort) [8]. This is a pregnancy cohort that recruited 3387 women at less than 25 weeks gestational age in Calgary, Canada. Initial recruitment took place from May 2008 and December 2010 [8]. Women completed one survey at < 25 weeks gestational age, one at 34–36 weeks gestational age, and one at four months postpartum [8]. Four more surveys were conducted when their child reached one year (2009–2012), three years (2012–2014), five years (2014–2016), and eight years of age (2017–2019). Finally, a survey was conducted during the COVID-19 pandemic between May 20 and July 15, 2020 [9, 10]. For the 8-year and COVID-19 surveys, data were collected and managed using Research Electronic Data Capture (REDCap) electronic data capture tools hosted at University of Calgary [11, 12], prior to REDCap, data were collected using physical surveys and TeleForm to scan and verify data. This study used the first survey at < 25 weeks gestational age as the baseline cohort and the 3-year follow-up to assess non-participation. Three-year follow-up is chosen as there was little loss to follow-up in this cohort during gestation and at birth; at the 1-year follow-up, there were administrative challenges that affected response rate, but for reasons not related to general attrition. STATA 16.0 statistical software (StataCorp, College Station, TX, USA) was used for all analyses while the ggplot2 package in R software [13] was used to generate figures.

### Model development

We examined two models: a priori and LASSO variable selection method, described below. For both models, the first survey was used to identify variables for inclusion in prediction models that ultimately led to weight development. The first survey included 127 variables across multiple topics, including socio-demographics, prenatal physical and mental health, lifestyle, and pre-pregnancy and life events. For both models, we used the follow-up survey conducted at three years for the outcome point (i.e., women who did not attend the 3-year survey were considered lost to follow-up). We later applied these models to subsequent waves of data collection (5-year follow-up, 8-year follow-up, and once during the COVID-19 pandemic).

The first method for weight generation followed a knowledge-based variable selection approach. Investigators with subject matter expertise in pregnancy cohorts (KA, SM, SP, TMP) met several times and collectively identified possible variables for inclusion, including possible interaction terms that could be related to drop-out over time. Decisions on which variables to include were based on existing content expertise as well as the quality of variable data (i.e., high proportion of missing data).

The second method followed a LASSO variable selection method described by Schmidt et al. [14]. This method was used to develop weights in a child cohort and uses least absolute shrinkage and selection operator (LASSO) regression to select relevant variables [15]. Categorical variables were left in categories as they were initially coded with the addition of a category for missing in some cases. For those categorical variables with missing data, missingness was recoded so that ‘missing’ became a category of the variable itself. If a single level within a categorical variable had a large coefficient based on the initial LASSO regression, we retained the overall categorical variable as a candidate for the next step of variable selection. This meant that continuous variables were cut into relevant categories and another level of “missing” was created. Next, we split the variables of interest into seven relevant context themes: Sociodemographic Characteristics, Pregnancy History, Conception History, Prenatal Care, Lifestyle and Health Care Use During Pregnancy, Mental Health/Social Support, and Smoking/Drug/Alcohol Consumption (current and previous). We applied LASSO regression with 10-fold cross validation to each context theme such that the tuning parameter minimizes the out-of-sample prediction error [14, 16]. The three variables with the largest coefficients from the LASSO regression were fit in a multivariable logistic regression model and area under the receiver operating curve (ROC), sometimes referred to as C-statistic, was calculated from predicted probabilities. One at a time, the variable with the next largest coefficient was fit to the model and this process was repeated until the ROC was not significantly different (p > 0.05) from the previous ROC. This was completed for each context theme and all variables from each context them served as the candidate variables for the final model. Next, all of those top contributing variables, based on coefficient size, from each context theme were combined into a larger LASSO model. Only non-zero coefficients were selected for inclusion in the final logistic regression model.

### Model assessment

We assessed the ability of the model to predict continuing participation through discrimination and calibration for both approaches. We assessed discrimination using area under the ROC. ROC plots are among the most common method of assessing discrimination and represents a curve of sensitivity over 1-specifictiy where sensitivity represents true positives (cases) while specificity represents true negatives (not cases) [17]. Values for ROC range from 0.5 (no better assessment than chance) to 1.0 (perfect discrimination). The following cut-offs are often suggested as guidelines to assess discrimination: ≤0.5 is no better than chance, > 0.5 and < 0.7 is poor, > 0.7 ≤ 0.8 is acceptable and > 0.8 is excellent [18]. Calibration of the model relates to the accuracy of predicted risk and has been defined as “for patients with a predicted risk of R%, on average R out of 100 should indeed suffer from the disease or event of interest” [19]. We assessed calibration through a combination of Hosmer-Lemeshow goodness-of-fit test, mean calibration, and calibration plots [20]. We then applied this model to the next wave of data collection (i.e., 5-year follow-up) to assess the temporal validity of the models and assess in the same way.

### Weights assessment

Using the models derived from the a priori and LASSO variable selection method, we calculated predicted probabilities and stabilized inverse probability weights [21]. We applied stabilized weights as they typically result in less variance than non-stabilized weights [21, 22]. The means, standard deviations (SDs), and ranges of the weights were calculated and plotted. Weights were truncated at the 0.5th and 99.5th percentiles to avoid bias due to extreme weights [23]. Weight performance was measured by comparing baseline characteristics of those who continued in the study and those who did not, with and without the weights. It has been suggested that the standardized difference is the preferred measure for comparing weight balance between groups (continued in study vs. lost to follow-up) and that a difference between groups of less than 10% is negligible [24]. We use the “pbalchk” package in STATA to calculate the standardized difference. Standardized differences can be calculated for continuous and categorical variables and involve both means for the continued and lost-to-follow up groups and their variances; for more information on this calculation see Austin, 2009 [25].

Finally, the same model identified at the 3-year follow-up was then re-fit to develop weights for each of the subsequent waves (i.e., 5-year follow-up, 8-year follow-up, and the first survey during the COVID-19 pandemic). The performance of these weights was assessed as above.

## Results

Based on the 3,351 singleton births in the All Our Families cohort, 1,990 (59.4%) continued participation at the three-year follow-up while 1,361 (40.6%) did not. Of note, the study population in follow-up waves differed slightly from baseline due to various reasons, such as child age eligibility for standardized developmental scales when data collection was initiated and associated funding and ethical constraints [9]. At the three-year follow-up 69% of participants from the two-year follow-up responded to the survey [9]. However, since some participants had dropped out at earlier waves (during pregnancy and at-birth waves), this represented 59% of the participants initially enrolled in the study. Ultimately, the a priori model contained 18 variables while the LASSO variable selection method model contained 22 (Table 1). The two models shared four variables (Education, Ethnicity, Physical Component Summary, and Previous History of Adverse Birth Outcomes).

**Table 1:** Variables included in participation models

Context Theme	a priori	LASSO variable selection method
Sociodemographic	Education	Education
	Ethnicity	Ethnicity
	Experienced Food Insecurity in Past Year	House Ownership
	Income	Paternal Age
	Maternal Age	
	Marital Status	
	Number in Household	
	Pre-Pregnancy BMI	
	Time In Canada	
Pregnancy History	History of Adverse Pregnancy Outcomes (i.e., stillbirth or miscarriage)	History of Adverse Pregnancy Outcomes
	Parity	Number of Previous Pregnancies
		Maternal Preterm Birth
		Mother of Participant was a Preterm Birth
Conception History	Used Conception Aids	Used Artificial Insemination
		Number of Fertility Aids Used
		Was Trying to Become Pregnant
		Received Advice on Conception from Health Professional
Prenatal Care	Difficulty Obtaining Prenatal Care	Number of Prenatal Health Care Visits
	First Prenatal Visit (gestational age)	Saw a Dentist in the Past Year
Pregnancy Lifestyle and Health Care Use	-	Fruit/Vegetable Consumption During Pregnancy
	-	Saw Health Care Provider After Finding Out About Pregnancy
Mental Health/Social Support	SF-12 Physical Component Score [26]	SF-12 Physical Component Score [26]
	Social Support Scale [27]	Partner Supportive of Pregnancy
	Reported History of Mental Illness	Perceived Stress Scale [28]
Smoking/Drug/Alcohol Consumption	History of Drug or Alcohol Dependance	Days per week of drug use (pre-pregnancy)
		Number of Alcoholic Drinks per day (pre-pregnancy)
		Number of Cigarettes per day (pre-pregnancy)

**Fig. 1:**
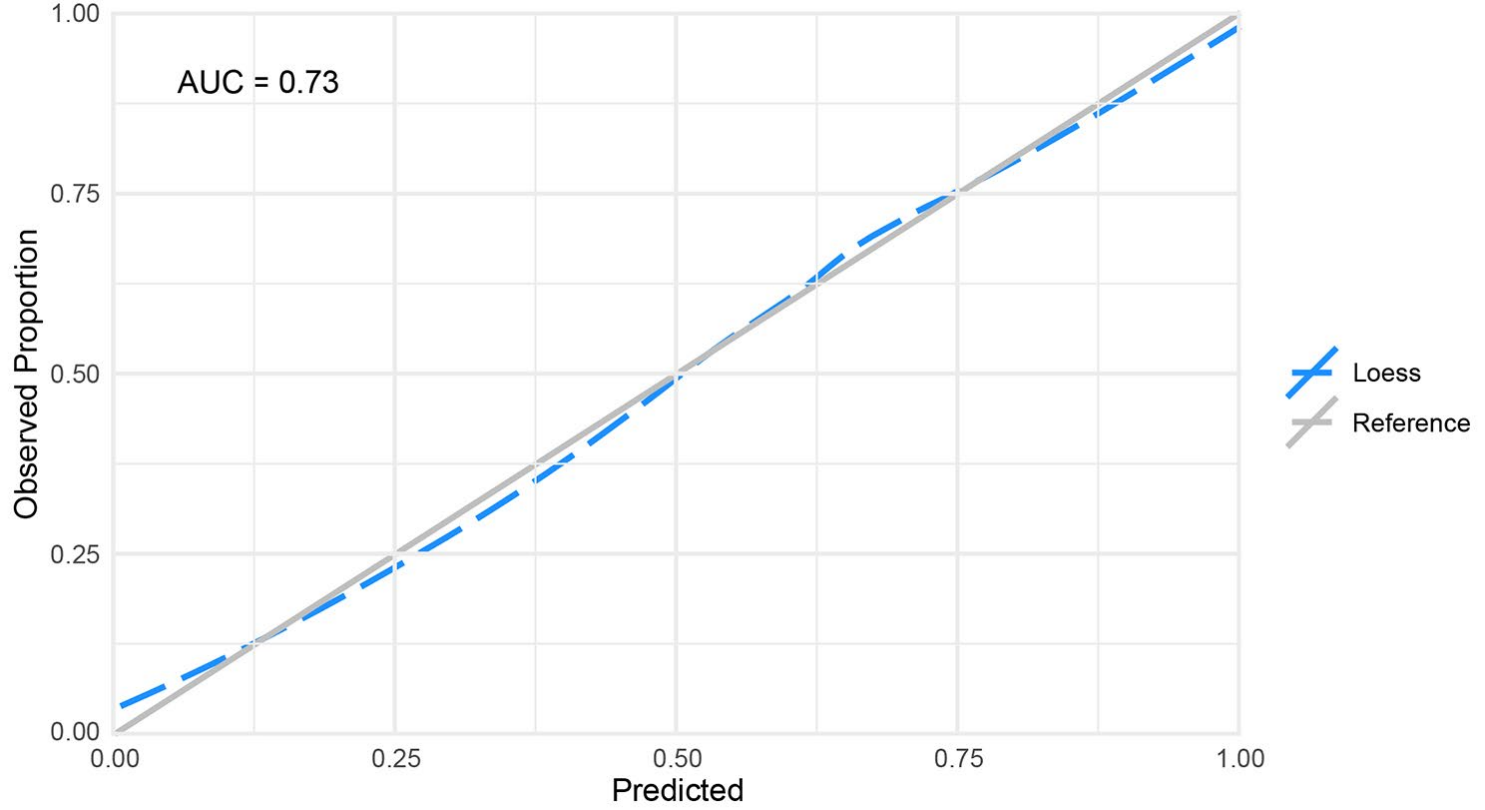
Results of Calibration Curve for LASSO variable selection method Model; AUC: Area Under the Receiver Operating Curve

**Fig. 2:**
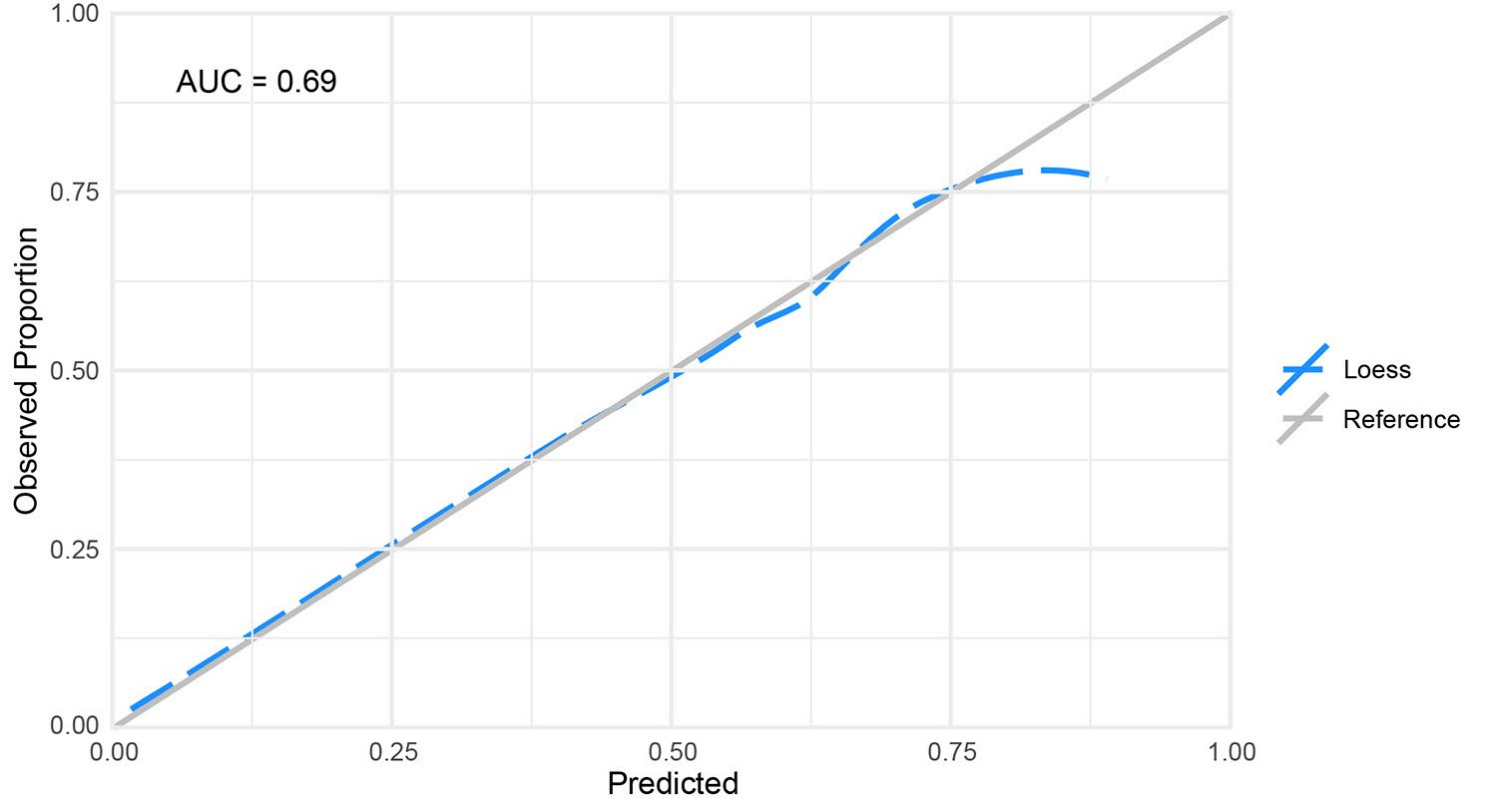
Results of Calibration Curve for a priori Model; AUC: Area Under the Receiver Operating Curve

The a priori model had poor-acceptable discrimination ROC of 0.69 (95% CI: 0.67–0.71) while the LASSO variable selection method model had acceptable discrimination ROC of 0.73 (95% CI: 0.71–0.75). Hosmer-Lemeshow goodness-of-fit tests with 10 bins were non-significant for both the knowledge-based (p = 0.329) and the LASSO variable selection method approach (p = 0.242). A statistically non-significant goodness-of-fit test indicates no statistical difference in the observed cases from the predicted cases [29]. A non-significant goodness-of-fit result implies a well-calibrated model; however, a goodness-of-fit test alone may not be sufficient to assess calibration [19]. To this end, we considered the mean calibration where “the average predicted risk is compared to the overall event rate” [19]. In this case, the ‘event rate’ is considered as the proportion of individuals who continue in the study at the 3-year follow-up and is compared with the calculated proportion derived from the a priori and LASSO variable selection method models. Mean calibrations were 0.594 (95% CI: 0.58–0.60) and 0.594 (95% CI: 0.59–0.60) for the a priori and LASSO variable selection method models, respectively, compared with an observed proportion of continued participation of 0.594. Given that the mean calibrations in the two models were very similar to that of the observed proportion, both models appeared well-calibrated. In addition, we visually examined the calibration plots for each model (Figs. 1 and 2). The a priori and LASSO variable selection method models were re-fit on the next wave of data collection (5-year follow-up) and performed similarly to the previous wave with ROC of 0.69 (95% CI: 0.67–0.71) and 0.73 (0.72–0.75), non-significant goodness-of-fit tests of p = 0.567 and p = 0.307, and mean calibration of 0.596 (95% CI: 0.59–0.60) and 0.593 (95% CI: 0.59–0.60), respectively.

In calibration plots, an ideal plot (a diagonal line with slope of 1 and intercept of 0) is presented with a calibration curve derived from the model data and demonstrates how similar (or not) the estimated risk is to observed risk. The plot is assessed by examining the curve slope (target of 1.0) and by using a loess function to compare curve of predicted risk with the ideal plot [18]. Both Hosmer-Lemeshow tests and mean calibration suggested moderate calibration, as did the calibration plots; although, the LASSO variable selection method model seemed more well-calibrated at higher values than the a priori model.

The stabilized weights for the a priori model had a mean (SD) of 1.00 (0.58) and a range of 0.43–10.1. The LASSO variable selection method model had a mean (SD) of 1.00 (0.74) and a range of 0.42–23.1. After trimming, the LASSO variable selection method and a priori models had maximum weights of 4.8 and 4.9, respectively. This resulted in changes to 33 individual’s weights in both models. As well, mean (SD) for the a priori and LASSO variable selection method models were 0.99 (0.46) and 0.99 (0.51), respectively.

### Weights performance

**Fig. 3:**
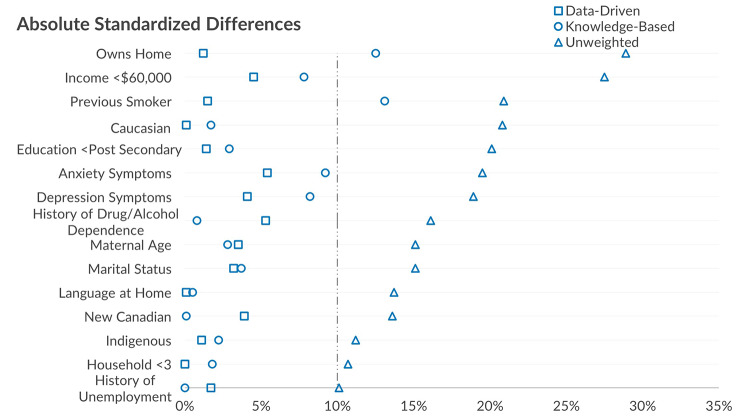
Comparing the unweighted absolute standardized differences with the stabilized truncated weights of a priori and LASSO variable selection method models

The absolute standardized differences were calculated across baseline demographic variables (chosen *a priori*) in the unweighted group were as large as 28.9% for home ownership and 27.5% for income (binary outcome split at $60,000) and a mean of standardized differences of 17.5%. In the a priori model, the largest absolute standardized difference was 13.1% (smoking history) with two variables having a standardized difference of 10% or greater and a mean of standardized differences of 4.6% (Fig. 3). In the LASSO variable selection method derived weights, the largest absolute standardized difference was just 5.4% (anxiety symptoms) with no variables greater than 10% and a mean of standardized differences of 2.5%. Comparisons of baseline characteristics are based on complete data at baseline; of the 15 variables measured, eight were missing data in ≤ 1% while the other seven (Income, Anxiety, Symptoms, Depression Symptoms, Maternal Age, New Canadian, and Household Size) ranged from 1 to 4.4%.

**Fig. 4:**
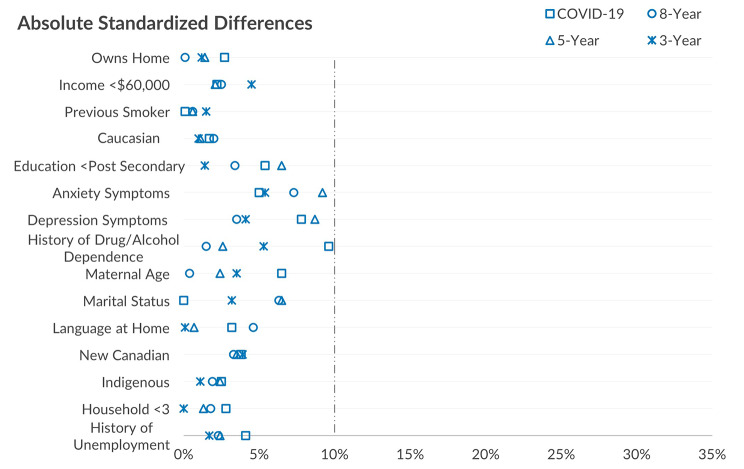
Comparing absolute standardized differences with the stabilized truncated weights derived from LASSO variable selection method model across data collection follow-up waves (3-, 5-, 8-, year follow-up and follow-up during COVID-19) in longitudinal cohort

Since the LASSO variable selection method weights appeared to perform better, weights were developed using this approach and applied to subsequent waves of data collection with performance evaluated in the same way (Fig. 4). Across each follow-up wave of data collection (3-year, 5-year, 8-year, and COVID-19 survey [approximately 12-years of follow-up]), absolute standardized differences remained below 10% for baseline demographic variables.

## Discussion

This study aimed to develop non-response weights for a pregnancy cohort that has followed participants for more than 12 years. To accomplish this, we examined two approaches: one a priori and another LASSO variable selection method. The LASSO variable selection method approach produced robust weights that addressed non-response bias more than the a priori approach. The data driven approach, however still required content knowledge in how data were grouped, combined, or split. These weights can be applied to analyses across multiple waves of data collection to reduce bias. While the a priori model performed well, the weights themselves did not reduce differences in baseline characteristics to the same degree as the LASSO variable selection method model. While the models contained different specific variables, there was some overlap in that variables between the models captured similar concepts. For example, the a priori model used the combined variable of ‘history of drug/alcohol dependence’ while the LASSO variable selection method model included drug use per week and number of alcoholic drinks per day. While both the a priori and LASSO variable selection method models had access to the same calculated variables, their component parts, and interaction terms, the a priori model attempted to create simplicity and reduce the number of variables within an overarching theme, the nuance of more specific variables was ultimately found to be more informative. As well, the a priori variable selection ended after initial selection of variables. Typically, in developing prediction models, the investigators would examine performance and re-calibrate as necessary, but for the purposes of variable selection performance this was not done. Further, the weights derived from the LASSO variable selection method approach were robust across waves. That is, the balance achieved at the 3-year follow-up was generally maintained through the 5-year and 8-year follow-up as well as through the survey during the COVID-19 pandemic (12 years after baseline). This indicates that the same factors influence retention over time, and that one model can be used to develop weights, and then applied consistently over several waves of data collection.

Unweighted differences in baseline characteristics existed with respect to attrition status during follow-up, suggesting the potential for selection bias. However, while bias due to attrition is possible in cohort studies, and should be considered, bias is not guaranteed simply due to differing baseline characteristics of those who continue those who drop-out if those differences do not exist between groups as they relate to the exposure-outcome relationship of interest. Previous work by this group has used weighted and unweighted results showed a slight difference in magnitude of results but no difference in trends [10]. Further, recent work has demonstrated no difference in results comparing modelled results of complete case analysis and inverse probability weighting using missing at random, missing not at random, and missing completely at random data [30]. To better understand the extent of bias due to attrition, comparison of analyses with and without weights is suggested. The weights created for this sample balanced demographic characteristics of those who continued participation and those who did not and serve as another way to quantitatively examine the potential role of attrition in creating bias in our longitudinal study cohort.

There exist some methodologic challenges in creating effective weights while also ensuring no undue influence of extreme weights. There is no clearly defined point at which to truncate weights but it is important to consider both heterogeneity in order to achieve balance and the role of extreme weights. The use of a very small amount of truncation seemed to be effective for this particular sample. By truncating just at the 99.5th percentile, we see the range in the LASSO variable selection method weights drop from 23.1 to 4.8 which would indicate just a few “outliers” that could have spuriously influenced the weights.

Strengths of this study include examining two approaches to developing weights and the comparison of the two. As well, this study used a large sample of over 3,351 participants with 127 individual variables that were considered. The breadth of variables allowed us to consider a multitude of factors that could predict continuation in the cohort in later waves.

This study is not without limitations. The LASSO variable selection method approach used missing data as a level within categories. This allowed us to maintain a large sample size, but it also meant that variables that would normally be continuous were categorized to create this missing level. Categorization of continuous variables can result in loss of information given the collapsing of participant data into groups.

This study outlined two approaches to developing non-response weights to address bias that may be introduced due to attrition, with a LASSO variable selection method approach creating weights performing better that a priori approach in balancing baseline characteristics. The All Our Families cohort observes approximately 60% of participants returning to the study eight years after giving birth, in line with other major pregnancy cohorts [31–33]. The use of inverse probability weights considers the potential effect of non-response bias and the weights developed here can be applied to future studies using the AOF cohort data in secondary analyses and subsequent data collections; a further advantage of the use of these weights is that they can be easily applied to a variety of outcome models (i.e., linear regression, logistic regression, survival analysis). Importantly, the approach used in the present study in creating these weights could be applied in other cohorts, where the potential for selection bias exists due to attrition. Balancing the characteristics of participants at later cohort data collection waves to the sample recruited at baseline increases the confidence that temporal associations better reflect the experience of the target population.

## Data Availability

The datasets analysed during the current study are not publicly available as they contain personal participant information but are available from the corresponding author, through the All Our Families Cohort Study, on reasonable request.
